# B-cell maturation antigen (BCMA) in multiple myeloma: rationale for targeting and current therapeutic approaches

**DOI:** 10.1038/s41375-020-0734-z

**Published:** 2020-02-13

**Authors:** Nina Shah, Ajai Chari, Emma Scott, Khalid Mezzi, Saad Z. Usmani

**Affiliations:** 1 University of California San Francisco, San Francisco, CA USA; 2 Mount Sinai Hospital, New York, NY USA; 3 GlaxoSmithKline, Portland, OR USA; 4 Amgen Inc, Thousand Oaks, CA USA; 5 Atrium Health, Charlotte, NC USA

**Keywords:** Medical research, Cancer

## Abstract

Despite considerable advances in the treatment of multiple myeloma (MM) in the last decade, a substantial proportion of patients do not respond to current therapies or have a short duration of response. Furthermore, these treatments can have notable morbidity and are not uniformly tolerated in all patients. As there is no cure for MM, patients eventually become resistant to therapies, leading to development of relapsed/refractory MM. Therefore, an unmet need exists for MM treatments with novel mechanisms of action that can provide durable responses, evade resistance to prior therapies, and/or are better tolerated. B-cell maturation antigen (BCMA) is preferentially expressed by mature B lymphocytes, and its overexpression and activation are associated with MM in preclinical models and humans, supporting its potential utility as a therapeutic target for MM. Moreover, the use of BCMA as a biomarker for MM is supported by its prognostic value, correlation with clinical status, and its ability to be used in traditionally difficult-to-monitor patient populations. Here, we review three common treatment modalities used to target BCMA in the treatment of MM: bispecific antibody constructs, antibody–drug conjugates, and chimeric antigen receptor (CAR)-modified T-cell therapy. We provide an overview of preliminary clinical data from trials using these therapies, including the BiTE® (bispecific T-cell engager) immuno-oncology therapy AMG 420, the antibody–drug conjugate GSK2857916, and several CAR T-cell therapeutic agents including bb2121, NIH CAR-BCMA, and LCAR-B38M. Notable antimyeloma activity and high minimal residual disease negativity rates have been observed with several of these treatments. These clinical data outline the potential for BCMA-targeted therapies to improve the treatment landscape for MM. Importantly, clinical results to date suggest that these therapies may hold promise for deep and durable responses and support further investigation in earlier lines of treatment, including newly diagnosed MM.

## Introduction

Multiple myeloma (MM) accounts for ~10% of all hematologic malignancies in the United States, with the highest incidences being observed in developed countries [1]. Considerable advances have been made in the last decade regarding the knowledge of the underlying biology and natural progression of MM. In addition, the use of proteasome inhibitors and immunomodulatory imide drugs (IMiDs) has improved treatment options for this condition [1]. Despite these advances, the 5-year survival rate for patients with MM is ~50% and can be lower in high-risk patients (e.g., frail elderly patients, MM with high-risk cytogenetics), highlighting an unmet need for improved treatment options for MM [1, 2]. With current approaches, MM is not considered curable and relapse is considered an inevitable part of the disease course, leading to the development of relapsed/refractory MM (RRMM) [1, 3–5]. Patients with RRMM have progressively shorter durations of remission and lesser responses to standard salvage therapies after relapse and treatment resistance. Of note, patients who progress within 18 months of starting initial therapy have particularly poor outcomes [1]. Ultimately, there remains an unmet need for novel therapies for newly diagnosed MM that could provide more durable responses than standard therapies, or even potentially a cure if used early in the disease course, as well as therapies for RRMM that can evade resistance to other therapies [1, 3, 4].

B-cell maturation antigen (BCMA) has emerged as a promising target for MM therapies. Currently, the three most common treatment modalities for targeting BCMA are bispecific antibody constructs including BiTE® (bispecific T-cell engager) immuno-oncology therapies, antibody–drug conjugates (ADCs), and chimeric antigen receptor (CAR)-modified T-cell therapy. In this review, we provide an overview of therapies from these classes that have presented or published clinical data, including the BiTE® molecule AMG 420, the ADC GSK2857916, and several CAR T-cell therapies including NIH CAR-BCMA, bb2121, and LCAR-B38M.

## Materials and methods

Published or presented clinical data for BCMA-targeted therapies were identified through PubMed (December 2, 2013 through May 16, 2019) and via search of abstracts from major oncology and hematology conferences (2016 through May 2019, up to and including ASCO 2019). BCMA-targeted therapies with clinical data presented or published as of May 16, 2019 are summarized in this review. The search terms used were “BCMA”, “CD269,” and “TNFRSF17” for the therapeutic target and “MM” and “myeloma” for the disease state. Major oncology and hematology conferences included American Society of Hematology, American Society of Clinical Oncology (ASCO), American Association for Cancer Research, European Hematology Association, International Myeloma Workshops, and Transplantation & Cellular Therapy Meetings (cosponsored by the American Society for Transplantation and Cellular Therapy and the Center for International Blood & Marrow Transplant Research). The most recent evidence regarding the biology of BCMA and its use as a biomarker was assessed using published research data and review articles.

## Rationale for targeting BCMA for treatment of MM

### Biology of BCMA

B-cell maturation antigen, also referred to as TNFRSF17 or CD269, is a member of the tumor necrosis factor receptor (TNFR) superfamily [6, 7]. Ligands for BCMA include B-cell activating factor (BAFF) and a proliferation-inducing ligand (APRIL), of which APRIL has a higher affinity for BCMA [8]. BCMA is expressed preferentially by mature B lymphocytes, with minimal expression in hematopoietic stem cells or nonhematopoietic tissue, and is essential for the survival of long-lived bone marrow plasma cells (PCs), but not overall B-cell homeostasis [9–12]. Membrane-bound BCMA can undergo γ-secretase–mediated shedding from the cell surface, leading to circulation of soluble BCMA (sBCMA) and reduced activation of surface BCMA by APRIL and BAFF [7, 13, 14].

#### Biology of BCMA in MM

The overexpression and activation of BCMA are associated with progression of MM in preclinical models and humans, which makes it an attractive therapeutic target [7, 15, 16]. Murine xenografts with induced BCMA overexpression grow faster than BCMA-negative controls. This overexpression leads to the upregulation of canonical and noncanonical nuclear factor kappa-B pathways, as well as enhanced expression of genes critical for survival, growth, adhesion, osteoclast activation, angiogenesis, metastasis, and immunosuppression [15]. Similar results are observed after APRIL-induced activation of BCMA in ex vivo human MM cells [15]. Furthermore, sBCMA can inhibit the activity of BAFF via complex formation, leading to MM-associated immunodeficiency [16]. BCMA is also expressed at much lower concentrations (9- to 50-fold lower) by plasmacytoid dendritic cells, which are known to help promote MM PC survival in the bone marrow environment [13, 17]. Additional details regarding the role of BCMA in B-cell biology and in MM, including illustrations, can be found in other reviews [18–21].

#### BCMA as a biomarker for diagnosis of MM

Malignant MM PCs typically compose a small subset of bone marrow cells, so accurate identification of these cells is important to ensure representative characterization of the disease [22]. The traditional MM biomarker CD138 is highly specific to PCs but rapidly disappears from the cell surface when sample analysis is delayed or if the sample is frozen [22]. Therefore, additional biomarkers to diagnose or monitor MM are needed.

BCMA is highly expressed on malignant PCs collected from patients with MM compared with normal bone marrow mononuclear cells (BMMCs) from healthy donors, and several studies have assessed whether BCMA has value as a marker for diagnosis, prognosis, and/or as a predictor of treatment response (Table 1) [7, 23–28]. In contrast with CD138, BCMA is readily identified in delayed and frozen MM samples [22]. The levels of membrane-bound BCMA can be measured by various techniques (e.g., flow cytometry, immunohistochemistry), with flow cytometry being more sensitive than immunohistochemistry, though the quantification of BCMA levels can differ between studies owing to differences in methodology [7, 23, 28]. Interestingly, BCMA mRNA is expressed at similar levels by malignant PCs in patients with newly diagnosed MM and RRMM, suggesting that BCMA may be a promising therapeutic target throughout the MM disease course [24].

**Table 1:** B-cell maturation antigen as a biomarker, prognostic marker, and predictor of response to treatment in humans.

Study	Methods	Results
Sanchez et al. [7]	• Measured surface BCMA and soluble BCMA levels using FC of samples collected from pts with ND and treated MM, pts with MGUS, and healthy controls • Assessed correlation of BCMA levels with objective response to anti-MM therapy, including PIs, IMiDs, and PLD	• Pts with ND MM (*n* = 50) had elevated surface BCMA expression and soluble BCMA levels compared with healthy controls • Previously treated pts with ≥PR (*n* = 80) had lower soluble BCMA levels than pts with progressive disease (*n* = 79) • Pts with BCMA levels above the median (*n* = 162) had a shorter OS than pts with BCMA levels below the median • Soluble BCMA levels did not correlate with use of specific anti-MM agents (e.g., PIs, IMiDs, PLD)
Lee et al. [25]	• BM aspirates collected from pts with ND or RRMM, assessed for BCMA expression by FC	• Primary MM cells varied in surface BCMA levels • In pts with sequential BM samples (*n* = 3), BCMA expression persisted throughout disease relapses after non-BCMA-targeted therapies (e.g., ASCT, chemotherapy), even in pts with low-level disease
Seckinger et al. [24]	• Malignant PCs collected from samples of previously untreated pts or pts with relapsed MM and assessed with multidimensional FC	• All MM CD138^+^ cells expressed BCMA RNA, with similar expression between pts with ND (*n* = 630) and RRMM (*n* = 82)
Ali et al. [26] Brudno et al. [27] NCT02215967	• Enrolled pts with MM with uniform BCMA expression by IHC or FC [26] • Median lines of therapy: 7 (interim results, *n* = 12), 9.5 (final results at highest dose level of anti-BCMA CAR^+^ T cells, *n* = 16) [26, 27] • 63% of pts treated at the highest dose level were refractory to their previous treatment regimen [27]	• 61% (52/85) of pts screened for the study had BCMA+ PC samples by IHC • Pretreatment surface BCMA expression was widely variable between pts [27] • Soluble BCMA decreased significantly in pts who responded to anti-BCMA CAR^+^ T-cell therapy but not in pts with no antimyeloma response (*n* = 16)
Friedman et al. [28]	• BM biopsies collected from pts with MM (*n* = 29) • BCMA expression assessed by IHC	• BCMA was expressed on all MM samples, though expression was variable • In 41% of MM BM biopsies, BCMA+ cells composed >50% of tumor area
Salem et al. [23]	• Pts with MM (*n* = 70) were screened for BCMA expression by FC • 39 samples assessed by both FC and IHC	• 94% (66/70) of pts were BCMA+ by FC • Among samples assessed by both FC and IHC, 38 were BCMA+ by FC and 28 were BCMA+ by IHC • BCMA expression was highly variable between samples

*ASCT* autologous stem cell transplantation, *BCMA* B-cell maturation antigen, *BM* bone marrow, *CAR* chimeric antigen receptor, *FC* flow cytometry, *IHC* immunohistochemistry, *IMiD* immunomodulatory drug, *MGUS* monoclonal gammopathy of undetermined significance, *MM* multiple myeloma, *ND* newly diagnosed, *OS* overall survival, *PC* plasma cell, *PI* proteasome inhibitor, *PLD* pegylated liposomal doxorubicin, *PR* partial response, *pts* patients, *RRMM* relapsed/refractory MM.

sBCMA levels are elevated in patients with MM and correlate with the proportion of MM cells in BMMC samples [7]. sBCMA may also serve as a valuable biomarker in select patient populations that are otherwise difficult to monitor. The levels of sBCMA are independent of renal function, which permits its use as a biomarker in patients with renal insufficiency, and sBCMA is detectable in the serum of patients with nonsecretory disease as well as in nonsecretory murine xenograft models [7, 21, 29].

#### BCMA as a tool for prognosis and treatment response

The clinical course of MM is variable and there remains a need for reliable methods to assess the prognosis of patients and monitor their disease status [29]. The levels of sBCMA have prognostic value, as patients with higher levels, particularly those ~25–325 ng/mL or higher, have poorer clinical outcomes than those with lower sBCMA values [7, 25, 29]. Similarly, baseline sBCMA levels have been suggested to be inversely correlated with future response to treatment [7, 30], though this correlation has not been observed in all studies [25, 31–34]. Higher sBCMA levels in patients with monoclonal gammopathy of undetermined significance or smoldering MM also appear to be associated with an increased risk of progression to MM [35].

The measurements of sBCMA may also be useful for monitoring patient response to ongoing therapy. Patients who have responded to therapy have reduced sBCMA levels compared with patients with progressive disease [7, 27]. Changes in sBCMA levels tend to correlate with the clinical status of patients with MM during anti-MM treatment, as well as tumor mass in preclinical models [7, 21, 26–29, 36, 37]. For example, one study found that patients with a complete response (CR) had lower sBCMA levels (median, 38.9 ng/mL) than patients with a partial or minimal response (median, 99.7 ng/mL) or nonresponsive disease (median, 195.3 ng/mL) [29]. Because sBCMA has a much shorter serum half-life (24–36 h) compared with M-protein (3–4 weeks), changes in sBCMA more rapidly reflect changes in disease status than M-protein levels and therefore may serve as a useful alternative and potentially more sensitive marker for monitoring disease status [20, 34]. Notably, sBCMA levels do not appear to change more significantly in response to one particular class of anti-MM therapy over others [7].

The efficacy and durability of anti-BCMA therapies may be particularly dependent on sBCMA levels. It has been demonstrated that sBCMA can bind to and interfere with anti-BCMA antibodies [38]. In this case, drugs that inhibit γ-secretase could enhance the efficacy of BCMA-targeted therapy by reducing shedding of BCMA from the cell surface and subsequent interference of BCMA-targeted therapies by sBCMA [20, 21, 38]. An additional approach could be to use anti-BCMA monoclonal antibodies (mAbs) with higher specificity for membrane-bound BCMA than sBCMA [39]. As it is currently unclear whether changes in membrane-bound or sBCMA levels during therapy could alter the long-term efficacy of anti-BCMA therapies, additional investigation into the relationship between baseline sBCMA and response to BCMA-directed therapies is warranted.

## Treatment modalities to target BCMA

Given the selective expression of BCMA on malignant PCs, several BCMA-targeted therapies have been developed with the aim of eradicating these malignant cells through distinct mechanisms. Current anti-BCMA therapies generally fall into one of three classes: bispecific antibody constructs, including BiTE® (bispecific T-cell engager) molecules, ADCs, and CAR T-cell therapy. In this section, we provide an overview of anti-BCMA therapies in these classes, focused on therapies with clinical data.

### Use of minimal residual disease measures in MM

In addition to impressive response rates by International Myeloma Working Group criteria, several BCMA-targeted therapies described below have demonstrated minimal residual disease (MRD)-negative status in heavily pretreated patients with RRMM [27, 34, 40, 41]. Minimal residual disease is defined as the presence of a small number of tumor cells after treatment that is below the level of detection using conventional morphologic assessments (e.g., stringent CR [sCR], CR). The precise definition of MRD negativity depends on the threshold and detection method used (e.g., flow cytometry, next-generation sequencing) [42, 43]. The use of MRD endpoints in clinical studies of hematologic malignancies has been increasing over time, and achieving MRD negativity is associated with better clinical outcomes [42, 44]. Even in cases in which patients achieve a CR by conventional measurements, patients who are MRD negative may have longer overall and progression-free survival (PFS) compared with patients who achieve a CR but are MRD positive [42, 43]. Therapies that help patients attain MRD-negative status along with deep morphological remission (i.e., CR) could ultimately lay the groundwork for achieving a cure for MM [42]. However, there are limitations to MRD measurements in the RRMM setting. First, the measurement and definition of MRD may not always be reproducible across studies, as techniques for assessing MRD differ in sensitivity and the cutoff used for defining MRD (e.g., 10^−4^, 10^−6^) have not yet been standardized [42, 43]. Second, MRD negativity cannot be directly interpreted as a cure, and some patients who do not achieve deep molecular remission still achieve long-term disease control [42]. Third, there are limited clinical data that have directly assessed the role of MRD in MM for guiding treatment decisions [42, 43]. Finally, the assessment of MRD in MM to date has been primarily in the newly diagnosed or maintenance setting; therefore, the role of MRD in RRMM prognosis or guidance of future treatment remains unclear [42].

### Bispecific antibody constructs

**Fig. 1:**
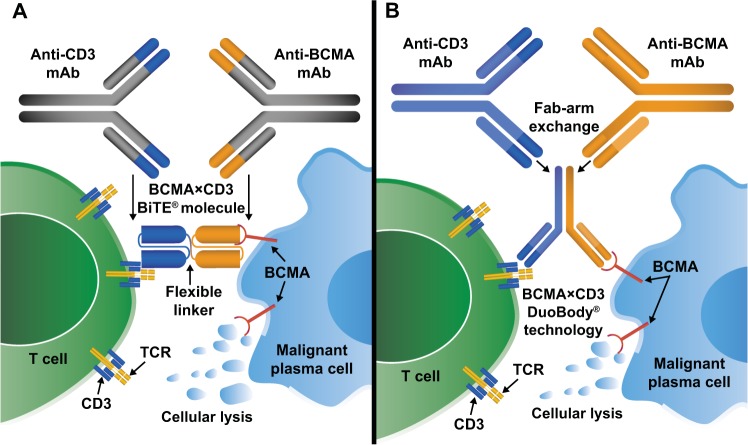
Bispecific antibody constructs facilitate cell-to-cell interactions via dual antigen specificity. Different forms of bispecific antibody constructs include BiTE® molecules (left) and DuoBody® technology (right). Engagement of T cells to malignant cells expressing B-cell maturation antigen (BCMA) leads to selective, redirected lysis of MM cells.

Bispecific antibody constructs are engineered to have dual antigen specificity to facilitate cell-to-cell interactions between the patients’ own T cells and malignant cells expressing tumor-specific antigens [45]. Several different structures have been used for bispecific antibody constructs investigated in oncological clinical trials, as illustrated in a recent review [46]. Forms of these constructs that have been investigated in MM include BiTE® (bispecific T-cell engager; Amgen, Thousand Oaks, CA, USA) molecules and DuoBody® (Genmab A/S, Copenhagen, Denmark) technology, among others. BiTE® molecules are fusion proteins consisting of single-chain variable fragments (scFv) with unique antigen specificities (Fig. 1) [45]. DuoBody® bispecific antibody constructs are generated via Fab-arm exchange, which uses mutations and recombination at the CH3–CH3 antibody interface to combine heavy and light chain homodimers from two separate mAbs into a single heterodimeric, bispecific antibody structure [47].

Of these two modalities, BiTE® molecules are currently the only type of bispecific antibody construct with preliminary efficacy data from clinical trials in MM [41, 48]. The rationale for use of BiTE® molecules in MM is also supported by the antitumor activity of blinatumomab, which is approved for treatment of select patients with acute lymphoblastic leukemia (ALL). Blinatumomab is a BiTE® molecule that engages CD3^+^ cytotoxic T cells and CD19^+^ B cells to recognize and eliminate CD19^+^ ALL blasts, leading to a survival benefit of 3.7 months compared with chemotherapy in patients with Philadelphia chromosome-negative B-cell ALL [49, 50]. BiTE® molecules for MM incorporate one scFv that engages the T-cell receptor CD3ε subunit, while the other engages a tumor-specific antigen expressed on malignant cells. This dual engagement leads to the formation of a cytolytic synapse between the T cell and the BCMA-expressing cell. Because formation of the cytolytic synapse is independent of standard antigen recognition and costimulation mediated by major histocompatibility complex class I, lysis of the target tumor cell occurs in a manner that is independent of immune escape mechanisms that tumor cells may develop to evade detection. CD3ε is expressed by all CD8^+^ and CD4^+^ T cells, which enables polyclonal T-cell activation, expansion, cytokine production, and tumor cell lysis [51].

#### AMG 420

AMG 420, formerly BI 836909, is a BCMA × CD3 BiTE® molecule that has been investigated in patients with RRMM (Table 2). Data from a first-in-human, phase 1 dose-escalation study (NCT02514239) reported an objective response rate (ORR) of 70% (7/10) at 400 μg/day, which included five MRD-negative CRs (i.e., a 50% MRD-negativity rate), one VGPR, and one PR [41, 48]. Minimal residual disease in this study was defined as <1 tumor cell per 10^4^ normal cells in the bone marrow by flow cytometry. As of cutoff for the most recently presented data, some responses were durable over 1 year, and two patients were in ongoing treatment at the 400 μg/day dose. Overall, median time to any response was 1 month. Serious AEs (SAEs) observed in more than one patient were infections and polyneuropathy (PN). Treatment-related SAEs included two grade 3 PNs and one grade 3 edema. Grade 2 or 3 cytokine release syndrome (CRS) was observed in 3 of 42 patients included in the phase 1 study. AMG 701, a half-life extended BiTE® molecule targeted to BCMA, appears to induce potent T cell-directed lysis of BCMA-positive MM cells in vitro [52] and is in clinical development.

**Table 2:** Clinical data for BCMA-targeted bispecific antibody constructs and ADCs.

Drug class	Name (sponsor)	Structure	Study design/patient population	Efficacy^a^	Safety
Bispecific antibody constructs	AMG 420 [41, 48] (Amgen)	BCMA × CD3 BiTE® (bispecific T-cell engager) molecule	• Phase 1^b^ (NCT02514239) • 6-week cycles (4 weeks continuous IV infusion, 2 weeks off) • Single-pt cohorts (0.2–1.6 µg/day) followed by cohorts of 3–6 pts (3.2–800 µg/day) • Pts with RRMM (≥2 lines of prior treatment); median of 5 prior lines • Median age: 65 years • Cytogenic risk: 33% high risk	• ORR (400 µg/day): 70% (5 CR, 1 VGPR, 1 PR) • **MRD–(400** **µg/day, 10 pts): 50% (all CR)** • Median time to any response: 1 month	• 800 µg/day not tolerable; 2/3 pts experienced DLTs (CRS, PPN) • Treatment-related serious AEs: 2 PNs, 1 edema • Grade 2–3 CRS in 3 pts
	PF-3135 [53] (Pfizer)	Humanized BCMA × CD3 bispecific antibody construct	• Phase 1 dose-escalation trial^b^ (NCT03269136) • Dose escalation with modified toxicity probability interval method • RRMM (treatment history: PI, IMiD, anti-CD38 mAb, alone or in combination)	ORR is a planned secondary outcome; efficacy data pending	• No DLT or CRS in first 5 pts dosed • 1 grade 3 ALT/AST elevation after cycle 1, day 1 infusion
ADCs	GSK2857916 [31, 61, 104] (GlaxoSmithKline)	Humanized IgG1 anti-BCMA mAb + MMAF	• Phase 1 two-part trial (NCT02064387) • Part 1 (38 pts): dose escalation (0.03–4.60 mg/kg IV Q3W, max 16 cycles) • Part 2 (35 pts): dose expansion • Pts with RRMM (treatment history: SCT, alkylators, PIs, IMiDs); 89% double refractory to PIs and IMiDs • Median age: 60 years • 14/35 pts received >5 prior lines of therapy • 8/35 pts had high-risk cytogenetics	• Clinical benefit rate (part 1): 25% (1 VGPR, 3 PR, 2 MR) • ORR (part 2): 60% (2 sCR, 3 CR, 14 VGPR, 2 PR) • Median PFS (part 2): 12.0 months	• 71% of pts experienced AEs that led to dose interruptions or delays • Most common AEs: thrombocytopenia, corneal events, cough • Most common grade ≥ 3 AEs: thrombocytopenia, anemia

*ADC* antibody–drug conjugate, *AE* adverse event, *ALT* alanine aminotransferase, *AST* aspartate aminotransferase, *BCMA* B-cell maturation antigen, *BiTE*^*®*^ bispecific T-cell engager, *CR* complete response, *CRS* cytokine release syndrome, *DLT* dose-limiting toxicity, *IgG* immunoglobulin G, *IMiD* immunomodulatory drug, *IV* intravenous, *mAb* monoclonal antibody, *MM* multiple myeloma, *MMAF* monomethyl auristatin F, *MR* minimal response, *MRD* minimal residual disease, *ORR* objective response rate, *PFS* progression-free survival, *PI* proteasome inhibitor, *PN* polyneuropathy, *PPN* peripheral PN, *PR* partial response, *pt* patient, *Q3W* every 3 weeks, *RRMM* relapsed/refractory MM, *sCR* stringent CR, *SCT* stem cell transplantation, *VGPR* very good PR.

^a^MRD data highlighted in bold.

^b^Data are from preliminary analyses of ongoing clinical trials.

#### PF-06863135

PF-06863135 (PF-3135) is a humanized bispecific IgG mAb consisting of anti-CD3 and anti-BCMA-targeting arms paired through hinge-mutation technology within an IgG2a backbone [53]. Safety results from a phase 1 dose-escalation study in patients with RRMM suggest that PF-3135 is well tolerated, with no dose-limiting toxicities or CRS events observed in the first five patients treated [53].

#### Other bispecific antibody constructs in clinical development

Other BCMA-targeted bispecific antibody constructs in clinical development that have demonstrated preclinical efficacy include JNJ-957 (a humanized BCMA × CD3 bispecific antibody construct with DuoBody® technology) [54], REGN5458 (a humanized BCMA × CD3 bispecific antibody construct) [55], TNB-383B (a fully human BCMA × CD3 bispecific antibody construct with a low-activating αCD3 arm that preferentially activates effector T cells over regulatory T cells) [56], and CC-93269 (previously known as BCMA-TCB2/EM901, a dual-arm, human IgG1-based bispecific antibody construct with one CD3 and two BCMA-binding sites) [57, 58].

### Antibody–drug conjugates

**Fig. 2:**
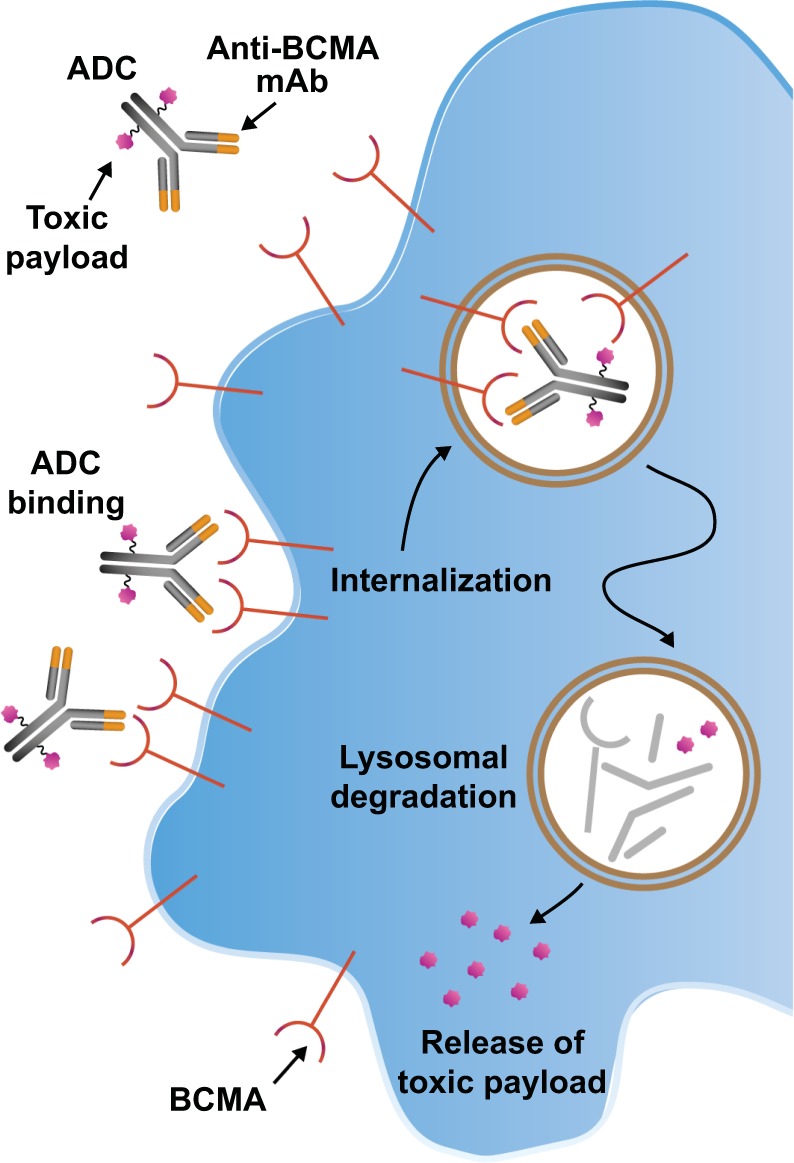
Antibody–drug conjugates bind to tumor-associated antigens on target cells, which leads to subsequent internalization and release of the toxic payload to induce selective cell death.

ADCs are tumor-associated antigen (TAA)-targeted mAbs conjugated to toxic payloads, such as tubulin polymerization inhibitor monomethyl auristatin F (MMAF), pyrrolobenzodiazepine (PBD), or the RNA polymerase II inhibitor α-amanitin, using a cleavable or non-cleavable linker [17, 31, 59, 60]. Once bound to TAA-expressing target cells, ADCs are internalized and the toxic payload is released to induce DNA damage and cell death (Fig. 2) [17, 39, 59]. Cleavable linkers are enzymatically processed within the target cell, while the action of ADCs with noncleavable linkers requires degradation of the attached antibody within lysosomes to release the payload [59]. Currently, one anti-BCMA ADC (GSK2857916) has demonstrated antimyeloma activity in a phase 1 trial (Table 2; described further below), and others have been investigated in preclinical species.

#### GSK2857916

The anti-BCMA ADC GSK2857916 consists of an afucosylated, humanized IgG1 anti-BCMA mAb conjugated to the tubulin polymerization inhibitor MMAF [31, 61]. The use of a defucosylated Fc region also helps facilitate the binding of effector cells to promote cell lysis of BCMA-expressing tumor cells via antibody-dependent cell-mediated cytotoxicity and antibody-dependent cellular-mediated phagocytosis [17]. GSK2857916 was investigated in a phase 1 trial of patients with progressive MM (NCT02064387) that included dose escalation and expansion (Table 2) [31, 61]. GSK2857916 was administered via 1-h infusions once every 3 weeks, and the ORR in the dose-expansion phase was 60% (21/35 patients), including two sCR, three CR, 14 VGPR, and two PR. Overall median PFS in these patients was 12.0 months. The most common grade 3 or 4 adverse events (AEs) during dose expansion were thrombocytopenia (34%) and anemia (17%). Corneal events were reported in 69% of patients, most of which were mild to moderate in severity, and had a median duration of 35 days. GSK2857916 was granted breakthrough therapy designation by the US Food and Drug Administration (FDA) in November 2017 and is currently being investigated in clinical trials in combination with IMiD therapies for treatment of patients with RRMM [62].

#### Other anti-BCMA ADCs in clinical development

Other anti-BCMA ADCs in clinical development include HDP-101 (an anti-BCMA antibody conjugated to the RNA polymerase II inhibitor amanitin), which may provide potent antitumor activity in patients with 17p deletions due to reduced RNA polymerase II subunit A expression in these patients, and MEDI2228, an anti-BCMA mAb conjugated to the PBD tesirine via a cleavable linker [39, 60, 63].

### Chimeric antigen receptor (CAR)-modified T-cell therapy

**Fig. 3:**
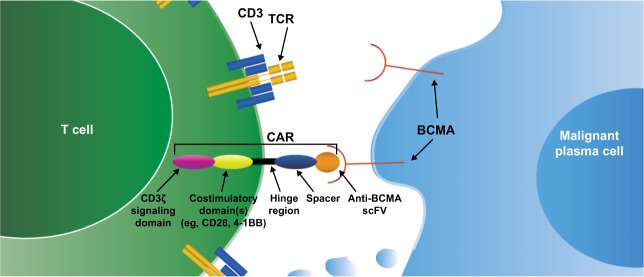
Chimeric antigen receptors (CARs) consist of tumor-associated antigen (TAA)-targeted single-chain variable fragments connected to intracellular signaling domains along with costimulatory domains. T cells that are genetically modified to express CARs bind to TAA-expressing target cells to initiate cellular lysis and death.

CAR T cells are genetically modified T cells that express a CAR targeted against a specific TAA, which upon binding initiates T-cell activation in a human leukocyte antigen–independent manner (Fig. 3) [64–67]. These CAR constructs consist of TAA-targeted scFvs (typically murine or human) connected to the CD3ζ intracellular signaling domain along with costimulatory domains (e.g., CD28, OX40, 4-1BB) by an extracellular spacer and transmembrane domain [65–67]. First-generation CARs only contained a CD3ζ signaling domain, but next-generation CARs have included multiple costimulatory domains to enhance the likelihood of CAR T-cell proliferation [65, 66]. Proliferation of CAR T cells in vivo has been shown to correlate with clinical activity and is frequently assessed in preclinical and clinical studies [26, 27, 68].

CAR T cells are typically generated from autologous T cells collected from the patient via leukapheresis, modified to express the CAR, and expanded ex vivo. While the CAR T cells are being manufactured, patients may receive bridging chemotherapy to maintain disease control before the CAR T cells are ready to be infused back into the patient [34, 64, 65, 69]. Before reinfusion of the expanded CAR T cells, most patients undergo a conditioning lymphodepletion chemotherapy regimen (e.g., fludarabine and cyclophosphamide), which reduces endogenous levels of lymphocytes to create a favorable environment for CAR T-cell expansion, persistence, and subsequent activity [64, 70, 71].

#### Anti-BCMA CAR T-cell therapies in clinical development

Several BCMA-targeted CAR T-cell therapies have demonstrated efficacy in early-phase clinical trials (Table 3). Though the constructs for these CAR T cells share some similarities, they differ in the costimulatory domains used (e.g., 4-1BB [33, 34, 37, 72, 73], CD28 [27, 74, 75], OX40 [75]), hinge regions (e.g., CD8 [27, 34, 37]), transmembrane domain (e.g., CD8 [27, 37, 76], CD28 [33, 74]), the species used to generate anti-BCMA scFvs (e.g., murine [27, 74], human [33, 37, 73, 77], llama [32]), and the use of modifications to enhance the safety of the CAR T-cell therapy (e.g., truncated epidermal growth factor receptor [73, 74, 77] or other safety switches [78]). The process of generating CAR T-cell therapies can also notably differ between different compounds, including the method of transduction (retroviral vs lentiviral), and the culture media used for ex vivo enrichment and stimulation of CAR T cells (e.g., paramagnetic beads coated with anti-CD3/anti-CD28 mAbs, OKT3, phosphoinositide 3 kinase inhibitors). Of note, although most CAR T-cell therapies to date are transduced using either a retroviral or lentiviral vector, the CAR T-cell therapy P-BCMA-101 is produced using the piggyBac^TM^ DNA modification system and is the only BCMA-targeted CAR T-cell therapy produced using a non-viral transduction method to date [78].

**Table 3:** Clinical data for BCMA-targeted CAR-modified T-cell therapies.

Name (sponsor)	Structure	Study design/patient population	Efficacy^a^	Safety
NIH CAR-BCMA, also referred to as anti-BCMA CAR or CAR-BCMA (NCI) [26, 27]	• Murine anti-BCMA scFv, CD28 costimulatory domain, CD8α hinge and transmembrane regions • Culture/Activation medium: anti-CD3 mAb and IL-2 • Transduction method: γ-retroviral vector	• Phase 1 dose-escalation trial (NCT02215967) • Lymphodepletion regimen^b^ before NIH CAR-BCMA infusion • Dose levels: 0.3, 1, 3, 9 × 10^6^ cells/kg • Measurable MM with uniform BCMA expression • 24 pts treated (16 pts received highest dose) • Median of 9.5 prior lines, 63% refractory to last treatment (at highest dose level) • 40% of evaluable pts at highest dose had high-risk cytogenetics	• ORR (9 × 10^6^/kg): 81% • **MRD–(9** **×** **10**^**6**^ **kg, 11 evaluated pts**^**c**^**): 100%** • Median event-free survival (9 × 10^6^/kg): 31 weeks • Peak CAR T-cell expansion occurred between 7 and 14 days post infusion for all pts • ≥1 blood CAR+ cells/μL detected between 26 and 57 days post infusion in 11 of 14 monitored pts	• Mild toxicity at lower doses • CRS-related toxicity substantial at 9 × 10^6^/kg • 38% of pts treated at 9 × 10^6^/kg required vasopressors for hypotension
Anti-BCMA CAR T cells with truncated EGFR safety switch (Henan University) [72]	• 4-1BB costimulatory domain, truncated EGFR as safety switch • Transduction: γ-retroviral vector	• Phase 1 trial^d^ (NCT03093168) • Lymphodepletion regimen^b^ before CAR T-cell infusion (9 × 10^6^ cells/kg) • RRMM (≥3 prior treatment regimens) • 20% BCMA expression on PCs	• ORR (7 evaluable pts): 86% (2 sCR, 2 VGPR) • **2 MRD–responses (both VGPR)** • CAR T-cell expansion and persistence were consistently observed	• No grade > 1 neurotoxicity or CRS observed at cutoff date
bb2121 [34] (Celgene)	• 4-1BB costimulatory domain • CD8α hinge and transmembrane domains • Culture/Activation medium: anti-CD3 and anti-CD28, OKT3 • Transduction: lentiviral vector	• Phase 1 two-part trial^d^ (NCT02658929) • Part 1: dose escalation (RRMM, ≥3 prior lines; ≥50% BCMA expression on PCs) • Part 2: dose-expansion (daratumumab experienced and refractory to last therapy, no BCMA expression required) • Lymphodepletion regimen^b^ before single bb2121 infusion • Dose levels: 50, 150, 450, 800 × 10^6^ cells • Median age: 60 years • Median prior lines of therapy: 7 • 45% of pts had high-risk cytogenetics	• ORR: 85% • Median DOR: 10.9 months • **MRD–(18 evaluable pts): 89%** • **MRD–(16 evaluable responders): 100%** • Median PFS: 11.8 months • 96%, 86%, 57%, and 20% of pts had detectable CAR T cells at 1, 3, 6, and 12 months, respectively	• Grade ≥ 3 AEs in 97% of pts • 76% of pts experienced CRS (6% grade 3) • Median time to CRS onset: 2 days • Median CRS duration: 5 days • 42% of pts experienced neurotoxicity, including 1 grade 4 event
bb21217 [40] (bluebird bio)	bb2121 structure with ex vivo culture addition of PI3K inhibitor bb007 to increase memory-like T-cell phenotype	• Phase 1 two-part trial^d^ (NCT03274219) • RRMM (≥3 lines of therapy, ≥50% BCMA expression on PCs) • Lymphodepletion regimen^b^ before bb21217 infusion • Planned dose levels: 150, 450, 800, 1200 × 10^6^ cells • Median age: 64 years • Median of 9 prior lines of therapy • 50% had high-risk cytogenetics	• ORR (7 evaluable pts): 86% (1 sCR, 3 VGPR, 2 PR) • **MRD–(3 evaluable responders): 100%** • 2/2 pts evaluable at 6 months had detectable CAR vector copies	• 5 of 8 pts experienced CRS (1 grade 3) • 1 pt experienced DLTs (grade 3 CRS, grade 4 encephalopathy with signs of PRES)
BCMA-CAR T cells [74] (Huazong University)	• Murine anti-BCMA scFv, CD8α hinge, CD28 transmembrane/costimulatory domain • Transduction: lentiviral vector	• Efficacy, safety, and tolerability trial (ChiCTR-OPC-16009113) • Lymphodepletion regimen^b^ followed by target dose of 5.4–25.0 × 10^6^ cells/kg • 28 pts (26 RRMM, 1 PCL, 1 POEMS)	• Strong BCMA expression (*n* = 22): ORR, 87% (73% CR); median DFS, 296 days • Weak BCMA expression (*n* = 6): ORR, 100% (33% CR or VGPR); OS, 206.5 days; median DFS, 64 days	• Grade 3 CRS occurred in 4 of 28 pts (14%)
BCMA CAR-T [105] (ShenZhen Biotechnology Company, Ltd)	Humanized alpaca anti-BCMA scFv, 4-1BB costimulatory domain	• Phase 1 trial in pts with RRMM^d^ (NCT03661554) • Lymphodepletion regimen followed by infusion of 2–10 × 10^6^ cells/kg • Average of 10 prior treatments	• ORR (28 days, 13 pts): 84.6% • ORR (10 weeks, 7 pts): 100% (3 sCR/CR, 1 VGPR, 3 PR) • Of 5 pts who reached 16 weeks, 4 kept remission and 1 relapsed	• 2 pts with grade 3–4 CRS (other pts had grade 0–2 CRS)
CART-19/BCMA [75] (The First Affiliated Hospital of Soochow University)	• OX40 and CD28 costimulatory domains, coinfusion with similar anti-CD19 CAR T cells • Culture/Activation medium: anti-CD3 mAb • Transduction: lentiviral vector	• Phase 1/2 trial^d^ (NCT03455972) • Pts received ASCT followed by coinfusion of CART-CD19 and CART-BCMA cells 14–20 days later • Pts with newly diagnosed stage III MM or pts who achieved ≤PR on prior therapy • All pts to date have >50% BCMA expression without CD19 expression on MM cells	• ORR (9 pts, after induction, ASCT, and CAR T-cell coinfusion): 100% (3 CR, 6 VGPR) • **MRD–(post-CAR T): 66.7%**	• Grade 1 or 2 CRS occurred in all 9 treated pts • No serious CRS or neurologic complications to date
CART-BCMA [37] (University of Pennsylvania–Novartis Alliance)	• Fully human anti-BCMA scFv, CD8 hinge and transmembrane domains, 4-1BB costimulatory domain • Culture/Activation medium: anti-CD3 and anti-CD28 paramagnetic beads and IL-2 • Transduction: lentiviral vector	• Phase 1 trial in 25 pts with RRMM (NCT02546167) • Cohort 1: 1–5 × 10^8^ CAR T cells • Cohort 2: Cy 1.5 g/m^2^ + 1–5 × 10^7^ CAR T cells • Cohort 3: Cy 1.5 g/m^2^ + 1–5 × 10^8^ CAR T cells • Administered via split infusion (3 days) • BCMA expression assessed but not required for eligibility • Median age: 58 years • Median of 7 prior lines of therapy • 96% of pts had high-risk cytogenetics	• Cohort 1 (9 pts): 1 sCR, 2 VGPR, 1 PR • Cohort 2 (5 pts): 1 PR • Cohort 3 (11 pts): 1 CR, 3 VGPR, 3 PR • Overall ORR: 48% • Median DOR: 124.5 days • Median PFS: 125 days (cohort 3) • Median OS: 502 days • Peak CAR T-cell expansion generally occurred 10–14 days post infusion • CAR T cells remained detectable in 100% (20/20) and 82% (14/17) of pts evaluated at 3 and 6 months post infusion, respectively	• Grade ≥ 3 AEs in 96% of pts • CRS in 88% of pts (grade 3–4: 32%) • Median time to CRS onset: 4 days • Median duration of CRS: 6 days • Neurotoxicity in 32% of pts, including 3 grade 3–4 encephalopathy • 1 grade 5 AE (death)
CT053 [106] (CARsgen Therapeutics)	Human anti-BCMA scFv, 4-1BB costimulatory domain	• Multicenter investigator-initiated study in 16 pts with RRMM • Pts must have ≥50% BCMA expression on malignant cells • Lymphodepletion regimen^b^ followed by single infusion of 0.5–1.8 × 10^8^ cells • Median age: 55 years • Median of 4 prior lines of therapy	• ORR (13 evaluable pts): 100% • 12/13 pts achieved PR+ within 4 weeks of infusion • Durable responses at data cutoff for 12/13 pts • 11/13 pts had notable persistence of CAR T cells up to 4–6 months post infusion	• No DLTs or neurotoxicity • Most common grade ≥ 3 AEs: thrombocytopenia, leukopenia, anemia, neutropenia, fever • 3 cases of CRS (1 grade 3)
CT103A [107] (Nanjing Iaso Biotherapeutics Co, Ltd)	• Fully human anti-BCMA scFv, CD8α hinge and transmembrane region, 4-1BB costimulatory domain • Transduction: lentiviral vector	• Dose-escalation trial in 9 pts with RRMM^d^ (ChiCTR1800018137) • 3 dose levels (1, 3, and 6 × 10^6^ cells/kg) • Median of 4 prior lines of therapy	• ORR: 100% • 2 pts with ongoing response at 120 days post infusion (1 CR, 1 PR) • Robust CAR T-cell expansion was observed even at the lowest dosage level	• At 1 or 3 × 10^6^ cells/kg, CRS cases were grade 0–2 • 1 DLT at 6 × 10^6^ cells/kg
FCARH143 [73] (Fred Hutchinson Cancer Research Center)	• Fully human BCMA scFv, 4-1BB costimulatory domain • Culture/Activation medium: anti-CD3/anti-CD28 paramagnetic beads (CD8^+^ and CD4^+^ cells cultured independently) • Transduction: lentiviral vector • Product infused in 1:1 ratio of CD4^+^ to CD8^+^ CAR T cells	• Phase 1 trial in pts with RRMM with ≥5% BCMA expression^d^ • Pts stratified into 2 cohorts by tumor burden • Lymphodepletion regimen followed by starting dose of 5 × 10^7^ EGFR + BCMA CAR T cells for each cohort • Median age: 63 years • Median of 8 prior regimens • All pts had ≥1 high-risk cytogenetic feature, 71% had ≥2 high-risk cytogenic features	• ORR (28 days, 6 evaluable pts): 100% • All pts surviving at median of 16 weeks of follow-up • CAR T cells remained detectable 90 days post infusion, representing ≤41.5% of CD3^+^ lymphocytes	• No DLTs • Grade ≤ 2 CRS in 6/7 pts • No neurotoxicity observed
JCARH125 [33] (Juno Therapeutics, Inc)	• Fully human anti-BCMA scFv, optimized spacer, CD28 transmembrane domain, optimized spacer, 4-1BB costimulatory domain • Transduction: lentiviral vector	• Phase 1/2 trial EVOLVE^d^ (NCT03430011) • Lymphodepletion regimen^b^ followed by JCARH125 infusion • Dose levels: 50, 150, or 450 × 10^6^ CAR T cells • Pts with RRMM (≥3 prior regimens) • Median age: 62 years • Median of 7 prior lines of therapy • 77% of pts had high-risk cytogenetics	• ORR (44 pts): 82% (48% ≥VGPR) • **MRD–(9 evaluable pts): 67%** • Trend toward increased persistence 2 months post infusion for doses ≥ 150 × 10^6^ CAR T cells	• CRS occurred in 80% of pts (grade ≥ 3: 9% of pts) • Median time to CRS onset: 3 days • Median duration of CRS: 5 days • Neurologic events in 25% of pts (grade ≥ 3: 7% of pts)
LCAR-B38M [32] (Nanjing Legend Biotech Co)	• 2 bispecific anti-BCMA variable fragments of llama heavy-chain murine Ab fused to 4-1BB signaling domain, CD8α hinge and transmembrane region • Culture/Activation medium: IL-2 • Transduction: lentiviral vector	• Phase 1 trial LEGEND-2 (NCT03090659) • Lymphodepletion regimen (cy alone) followed by LCAR-B38M (split into 3 infusions over 7 days) • Median LCAR-B38M dose: 0.5 × 10^6^ cells/kg • Pts with RRMM (median of 3 prior lines of therapy) • Median age: 54 years	• ORR (57 pts): 88% (39 CR, 3 VGPR, 8 PR) • **MRD–(57 pts): 63%** • Median PFS: 15 months • Median DOR: 14 months • Median OS: not reached	• Most common AEs: pyrexia (91%), CRS (90%), thrombocytopenia (49%), leukopenia (47%) • Most common grade ≥ 3 AEs: leukopenia (30%), thrombocytopenia (23%), AST increases (21%) • Median time to CRS onset: 9 days • Median duration of CRS: 9 days
MCARH171 [68, 77] (Poseida Therapeutics, Inc)	• Human-derived, 4-1BB costimulatory domain, CD8α hinge and transmembrane region, truncated EGFR safety system • Culture/Activation medium: phytohemagglutinin or CD3/CD28 beads in presence of IL-2 • Transduction: retroviral vector	• Phase 1 dose-escalation trial^d^ • Lymphodepletion regimen^b^ followed by MCARH171 infusion in 1–2 split doses • Mean doses (by cohort): 72 × 10^6^, 137 × 10^6^, 475 × 10^6^, or 818 × 10^6^ cells • Pts with RRMM (median of 6 prior lines of therapy) • 82% of pts had high-risk cytogenetics	• ORR (11 pts): 64% • ORR (dose 450 × 10^6^ cells, 5 pts): 100% • Median DOR: 106 days • Expansion and persistence of CAR T cells were dose dependent	• No DLTs reported • CRS occurred in 60% of evaluable pts (grade 3 in 20% of pts) • No grade ≥ 3 neurotoxicity
P-BCMA-101 [78] (Poseida Therapeutics, Inc)	• Anti-BCMA Centyrin™ fused to CD3ζ/4-1BB signaling domain, safety switch and selection gene • Transduction: piggyBac™ DNA modification system	• Phase 1 dose-escalation trial^d^ (NCT03288493) • Lymphodepletion regimen^b^ followed by P-BCMA-101 infusion • Dose range: 48–430 × 10^6^ cells (across 3 cohorts) • Pts with RRMM (≥3 prior lines) • 64% of pts had high-risk cytogenetics	• ORR (6 pts treated above cohort 1 doses): 83% (3 PR, 1 VGPR, 1 sCR) • CAR T-cell expansion peaked at 2–3 weeks and remained detectable at 3 months in all 3 evaluable pts	• No neurotoxicity or DLTs related to treatment • 1 pt (8%) developed grade 2 CRS • Most common grade ≥ 3 AEs: cytopenia, febrile neutropenia

*Ab* antibody, *AE* adverse event, *ASCT* autologous stem cell transplantation, *BCMA* B-cell maturation antigen, *CAR* chimeric antigen receptor, *CR* complete response, *CRS* cytokine release syndrome, *cy* cyclophosphamide, *DFS* disease-free survival, *DLT* dose-limiting toxicity, *DOR* duration of response, *EGFR* epidermal growth factor receptor, *IL* interleukin, *IV* intravenous, *mAb* monoclonal Ab, *MM* multiple myeloma, *MR* minimal response, *MRD* minimal residual disease, *ORR* objective response rate, *PC* plasma cell, *PCL* PC leukemia, *PD* progressive disease, *PFS* progression-free survival, *PI* proteasome inhibitor, *POEMS* polyneuropathy, organomegaly, endocrinopathy, monoclonal protein, skin changes, *PR* partial response, *PRES* posterior reversible encephalopathy syndrome, *pt* patient, *Q3W* once every 3 weeks, *RRMM* relapsed/refractory MM, *scFv* single-chain variable fragment, *sCR* stringent CR, *SD* stable disease, *VGPR* very good PR.

^a^MRD data highlighted in bold.

^b^Lymphodepletion regimen consisted of cy and fludarabine.

^c^Five patients not evaluated for MRD (three because of clinical lack of response, one because of baseline MRD negativity, one because of patient noncompliance).

^d^Data are from preliminary analyses of ongoing clinical trials.

In addition to differences in the structure and manufacturing of CAR T-cell constructs, clinical trial designs and results have differed between BCMA-targeted CAR T-cell therapies to date, including differences in the studied patient populations, dosing and persistence of CAR T cells, and efficacy and safety data (Table 3). Clinical data for several of these therapies show ORR > 80% in patients with RRMM. The most common AEs across therapies are CRS and neurotoxicity, though incidence, severity, and time to CRS onset vary by therapy.

#### bb2121 and bb21217

The CAR T-cell therapy bb2121 has been investigated in patients with RRMM who have ≥50% BCMA expression on malignant cells [34]. The ORR was 85% (28/33 patients) and 45% of patients experienced CR or sCR, with a median duration of response of 10.9 months. Median PFS was 11.8 months. In 16 responders evaluated for MRD negativity, 100% were MRD negative at 10^−4^ cells or better, 94% were MRD negative at 10^-5^ cells or better, and 19% were MRD negative at 10^−6^ cells. In contrast, two patients who did not achieve a response to bb2121 were MRD positive 1 month post infusion. All 33 patients experienced AEs, with 97% of patients experiencing at least one grade ≥ 3 AE. CRS occurred in 76% of patients, including grade 3 CRS in two patients. Among 14 patients experiencing neurotoxicity, one patient had grade 4 neurotoxicity 11 days after infusion. On the basis of early clinical data, bb2121 received breakthrough therapy designation from the FDA in late 2017.

Another CAR T-cell construct similar to bb2121, known as bb21217, is also under clinical investigation [40]. These CAR T cells are cultured in the presence of the phosphoinositide 3 kinase inhibitor bb007 ex vivo to promote a memory-like phenotype, which is hypothesized to increase the persistence and potency of CAR T cells. Among seven treated patients, ORR was 86% (one sCR, three VGPR, and two PR), and all three evaluable responders were MRD negative by next-generation sequencing. CRS was observed in 62.5% (5/8) of patients, including one case of grade 3 CRS that was accompanied by grade 4 encephalopathy with signs of posterior reversible encephalopathy syndrome.

#### NIH CAR-BCMA

NIH CAR-BCMA has been investigated in a phase 1 dose-escalation trial in patients with measurable MM and uniform BCMA expression on PCs [26, 27]. Among 16 patients treated with doses of 9 × 10^6^ cells/kg or higher, the ORR was 81% (13/16), and all 11 evaluated patients had MRD-negative disease 2 months after NIH CAR-BCMA infusion as assessed by bone marrow flow cytometry (limit of detection, 7 × 10^−6^). Duration of myeloma responses ranged from 2 to 51 weeks, and 6 of the 11 patients who were MRD negative had an ongoing response at the last follow-up before publication. Treatment-related toxicity was mild at lower doses (no grade ≥ 3 CRS). However, CRS-related toxicity was substantial at the highest dose tested (9 × 10^6^ cells/kg), particularly for patients with high tumor burden, and, overall, 38% of patients required vasopressor support for hypotension. Neurologic toxicities accompanying severe CRS were limited to confusion or delirium, except for one patient who experienced encephalopathy and muscle weakness consistent with PN.

#### FCARH143

FCARH143 is a fully human BCMA-targeting CAR T-cell therapy that is formulated in a 1:1 ratio of CD4^+^ to CD8^+^ CAR T cells for infusion and expresses a truncated non-functional human epidermal growth factor receptor to help identify transduced T cells [73]. Preliminary results from an ongoing phase 1 trial in patients with RRMM indicated that treatment with FCARH143 was associated with an ORR of 100% at 28 days in 6 evaluable patients, and all 6 patients had no detectable abnormal bone marrow PCs by immunohistochemistry and flow cytometry. All patients were currently alive at a median (range) of 16 (2–26) weeks of follow-up. Grade 2 or lower CRS was experienced by 86% of patients and no neurotoxicity was observed.

#### LCAR-B38M

LCAR-B38M is a dual epitope-binding CAR T-cell therapy directed against two distinct BCMA epitopes that was investigated in a phase 1 trial in patients with RRMM [32]. Treatment with three infusions of LCAR-B38M administered over 7 days resulted in an ORR of 88% (50/57 patients), including 39 CR, three VGPR, and eight PR, and an MRD negativity rate of 63% (36/57 patients) as assessed by bone marrow flow cytometry, defined as <1 tumor cell per 10^4^ normal cells. At data cutoff before publication, 20% of patients who achieved a PR or better had subsequently progressed. Median PFS was 15 months. The most common grade ≥ 3 AEs were leukopenia (30%), thrombocytopenia (23%), and aspartate aminotransferase elevations (21%). Ninety percent (51/57) of patients experienced CRS of any severity, including four patients (7%) with grade ≥ 3 CRS, and grade 1 neurotoxicity was observed in one patient. Similar efficacy and safety were observed in an additional exploratory trial of LCAR-B38M at a separate site with 17 patients with RRMM, regardless of whether LCAR-B38M was administered as a three-infusion or single-infusion process [79].

#### JCARH125

JCARH125 is a fully human CAR T-cell therapy with a 4-1BB costimulatory domain that has been investigated in a multicenter phase 1/2 trial in patients with RRMM (EVOLVE) [33]. Among 44 patients treated at doses of 50, 150, or 450 × 10^6^ cells, ORR was 82%, with 48% of patients achieving VGPR or greater. Some patients had improved responses over time, and six of nine evaluable patients were MRD negative by next-generation sequencing (defined as ≤1 tumor cell per 10^5^ normal cells) at day 29 post infusion. CRS occurred in 80% of patients and 9% experienced grade ≥ 3 CRS. Grade 1 to 2 and grade ≥ 3 neurotoxicity occurred in 18 and 7% of patients, respectively.

#### MCARH171

MCARH171 is a human-derived CAR T-cell therapy with a truncated EGFR safety system that has been investigated in a phase 1 dose-escalation trial [77]. In 11 patients, ORR was 64% across all dose levels tested; all five patients who received the higher dose levels tested (≥450 × 10^6^ cells) achieved an objective response. Responses ranged in duration from 17 to 235 days, with three of five patients treated at the highest doses having responses longer than 6 months and two patients having ongoing responses at 7.5 and 10 months of follow-up. Grade 1–2 and grade 3 CRS occurred in 40% and 20% of patients, respectively, and one case of grade 2 neurotoxicity (encephalopathy) was reported.

#### CART-BCMA

CART-BCMA is a CAR T-cell therapy with a fully human scFv with a 4-1BB costimulatory domain that has been investigated in a phase 1, open-label study in patients with RRMM [37]. Twenty-five patients were treated across three dose cohorts, which varied in CART-BCMA dose level and/or coadministration of cyclophosphamide (Table 3). The ORR across all 25 treated patients was 48% and was higher (55%) in those receiving the higher dose level (1–5 × 10^8^ CART-BCMA cells). The median (range) duration of response was 124.5 (29–939+) days. Three patients remained progression free at data cutoff, with a median overall survival of 502 days among all treated patients. Grade 3 or higher AEs were observed in 96% (24/25) of patients, regardless of attribution to study drug. CRS was observed in 88% of patients (32% grade 3 or 4), and 32% of patients experienced neurotoxicity (including 3 cases of grade 3–4 encephalopathy).

## Discussion and future perspectives

BCMA is a promising novel target for antimyeloma therapies. Different classes of BCMA-targeting drugs, including bispecific antibody constructs, ADCs, and CAR T-cell therapies, have shown antimyeloma activity in patients with RRMM and could help address a critical unmet need for therapies in patients with MM [1, 3, 4]. While there are not yet trials underway using BCMA-targeted therapies for treatment of newly diagnosed MM, these therapies could offer promise in this population as well, as supported by the high MRD negativity rates, high ORR, and durable responses reported to date with select BCMA-targeted therapies. As MRD negativity is associated with prolonged remission, further study is warranted to investigate whether BCMA-targeted therapies could provide durable responses or even a cure in earlier lines of therapy for MM, including newly diagnosed MM [42, 80].

Each BCMA-targeted treatment modality carries potential strengths and limitations. Bispecific antibody constructs are off-the-shelf therapies that have the potential to be available to patients to initiate treatment immediately and do not depend on ex vivo manipulation of patients’ cells. Clinical and notable antimyeloma activity has been observed with the BiTE® molecule AMG 420 in a phase 1 trial [41, 48]. One limitation of AMG 420, and similar bispecific antibody constructs, is that its relatively short half-life necessitates prolonged intravenous infusion using a central venous access device, though this short half-life may help manage treatment-emergent AEs, such as CRS [41, 45]. To address this limitation, several groups are developing bispecific antibody constructs with longer half-lives that are being investigated in ongoing clinical trials, including AMG 701 (NCT03287908) [52], CC-93269 (NCT03486067) [57], JNJ-64007957 (NCT03145181) [54], REGN5458 (NCT03761108) [55], and TNB-383B (NCT03933735) (Table 4) [56]. Unlike CAR T-cell therapies, bispecific antibody constructs themselves do not proliferate but rather act by inducing expansion of antigen-experienced T cells. Although it is unclear how to directly compare the immune expansion capability of bispecific antibody constructs and CAR T-cell therapies, it has been noted that the expansion of antigen-experienced T cells by bispecific antibody constructs can be order of magnitudes lower than the self-expansion of CAR T-cell therapies [81]. Because the resolution of malignant disease could require continued action of T cells over prolonged time periods, differences in T-cell expansion and persistence between bispecific antibody constructs and CAR T-cell therapies could lead to differences in durability of remission, though there is currently insufficient clinical data for BCMA-targeted therapies to date to make direct comparisons [81].

**Table 4:** Ongoing clinical trials of BCMA-targeted bispecific antibody constructs with extended half-lives^a^.

Name (sponsor)	Study design	Inclusion criteria	Outcome measures	Estimated completion dates^a^
AMG 701 (Amgen)	• Phase 1, open-label, dose-escalation and expansion study (NCT03287908) • Safety and tolerability of weekly IV infusions of AMG 701 will be evaluated during dose escalation, followed by expansion to assess efficacy and safety • Estimated enrollment: 135 pts	• Adult pts with RRMM after ≥2 lines of prior therapy (must include a PI, IMiD, or a CD38-directed cytolytic Ab; pts refractory to or intolerant of these therapies are eligible) • Measurable disease per IMWG criteria • ECOG PS ≤ 2	• Primary: incidence of AEs (48 months) and DLTs (28 days) • Secondary: antitumor activity measured by sCR, PFS, CR, VGPR, PR, and OS (48 months); PK (12 weeks)	• Primary: Jan 2021 • Study: Jul 2025
CC-93269 (Celgene)	• Phase 1, open-label, dose-escalation and expansion study (NCT03486067) • Safety and tolerability of escalating doses of IV infusion of CC-93269 (28-day cycle) will be evaluated during dose escalation, followed by expansion to further evaluate efficacy and safety • Estimated enrollment: 120 pts	• Adult pts with RRMM who have failed treatment with, are intolerant to, or are not candidates for available therapies for RRMM • Measurable disease • ECOG PS ≤ 1	• Primary: incidence of AEs and DLTs, non-tolerated dose, maximum tolerated dose (48 months) • Secondary: ORR (PR + VGPR + CR + sCR) per IMWG criteria; TTR, DOR, PFS, OS, PK, immunogenicity, tumor sensitivity/resistance (48 months)	• Primary: Jul 2021 • Study: Jun 2022
JNJ-64007957 (Janssen)	• Phase 1, open-label, dose-escalation and expansion study (NCT03145181) • Safety, tolerability, PK, and preliminary antitumor activity of JNJ-64007957 and identification of RP2D(s) • Estimated enrollment: 160 pts	• Adult pts with RRMM who have failed treatment with or are intolerant to established MM therapies (prior lines must include PI and IMiD in any order) • Measurable disease • ECOG PS ≤ 1	• Primary: DLTs (28 days), incidence of AEs (6 months) • Secondary: PK, immunogenicity, biomarker assessment (8 weeks), preliminary antitumor activity at RP2D(s) (end of treatment, ~91 days)	• Primary: May 2020 • Study: Sep 2021
REGN5458 (Regeneron)	• Phase 1/2, open-label study (NCT03761108) • Phase 1: assess safety, tolerability, and DLT and determine RP2D • Phase 2: preliminary antitumor activity of REGN5458 • Estimated enrollment: 56 pts	• Adults with RRMM who have failed, are intolerant to, or refused all therapeutic options, including either ≥3 lines of therapy including a PI, IMiD, and anti-CD38 Ab or progression on or after an anti-CD38 Ab with MM that is double refractory to a PI and an IMiD • Measurable disease • ECOG PS ≤ 1	• Primary: incidence of DLTs (28 days), incidence and severity of TEAEs, severity of AESIs (30 days after last dose), ORR per IMWG criteria in phase 2 (14 months after last dose) • Secondary: PK (64 weeks), immunogenicity, DOR, PFS, MRD-negative status, OS, incidence/severity of TEAEs, incidence/severity of AESIs, ORR in phase 1 (14 months after last dose)	• Primary: Dec 2022 • Study: Dec 2022
TNB-383B (Teneobio)	• Phase 1/2, open-label, dose-escalation and expansion study (NCT03933735) • Safety, clinical pharmacology, and clinical activity of TNB-383B • Estimated enrollment: 72 pts	• Adults with RRMM who have received ≥3 prior lines of therapy with exposure to PI, IMiD, and anti-CD38 Ab • Measurable disease • ECOG PS ≤ 2	• Primary: incidence of DLTs (21 days), incidence of AEs and SAEs (90 days), PK (12 weeks) • Secondary: immunogenicity, ORR (CR + PR per IMWG criteria), DOR (48 months)	• Primary: Mar 2021 • Study: Dec 2021

*Ab* antibody, *AE* adverse event, *AESI* AE of special interest, *BCMA* B-cell maturation antigen, *CR* complete response, *DLT* dose-limiting toxicity, *DOR* duration of response, *ECOG* Eastern Cooperative Oncology Group, *IMiD* immunomodulatory drug, *IMWG* International Myeloma Working Group, *IV* intravenous, *MM* multiple myeloma, *MRD* minimal residual disease, *ORR* objective response rate, *OS* overall survival, *PFS* progression-free survival, *PI* proteasome inhibitor, *PK* pharmacokinetics, *PR* partial response, *PS* performance status, *pt* patient, *RP2D* recommended phase 2 dose, *RRMM* relapsed/refractory MM, *SAE* serious AE, *sCR* stringent CR, *TEAE* treatment-emergent AE, *TTR* time to response, *VGPR* very good PR.

^a^As of October 23, 2019.

Similar to bispecific antibody constructs, ADCs do not require sample collection from the patient to generate a personalized ADC, and the antimyeloma activity of GSK2857916 has been observed in patients with RRMM [31]. The most common AEs observed with GSK2857916, thrombocytopenia and corneal events, are consistent with the known adverse effects of the toxic payload MMAF [31]. Indeed, the safety profile of ADCs depends on the toxic payload used. For certain ADC constructs, extracellular cleavage of the ADC before target cell penetration could lead to premature liberation of the toxic payload and negative effects on healthy cells, but the use of noncell-permeable payloads (e.g., MMAF) or non-cleavable linkers can reduce this concern [17, 59]. Similar to bispecific antibody constructs, ADCs can induce immunogenic responses against myeloma cells, which could help promote durable endogenous antimyeloma activity [17, 31]. However, similar to bispecific antibody constructs and in contrast with CAR T-cell therapies, ADCs are not anticipated to expand and persist in vivo based on their mechanism of action. This contrast may lead to differences in durability of responses compared with CAR T-cell therapies, though there have been no direct comparisons of BCMA-targeted ADCs and CAR T-cell therapies to date.

Early-phase clinical efficacy has been observed with several different anti-BCMA CAR T-cell constructs (Table 3). A notable advantage of CAR T cells is that these cells can expand after a single infusion, which may lead to persistent immunity against cancer cells [4, 64, 71]. The most common toxicities associated with CAR T cells include CRS and neurologic toxicity, which are typically managed with an IL-6 receptor antagonist (e.g., tocilizumab) and systemic corticosteroids, respectively [4, 66, 71, 82]. Other common toxicities include cytopenias and hypogammaglobulinemia [82]. One approach to avoid potential toxicities has been to engineer an “off switch” into CAR T-cell therapies so that the activity of these cells can be modified post infusion by dosing with an antibody-based switch [83]. Moreover, patients receiving CAR T-cell therapy may receive treatment with bridging chemotherapy before infusion, which could impact subsequent outcomes [69]. One limitation of CAR T-cell therapy is the prolonged manufacturing time needed before treatment, as several days to weeks are required for the collection of leukocytes from patients, ex vivo expansion and transduction of autologous T cells with CAR, and infusion at a specialized treatment center [27, 31, 71, 84]. This prolonged manufacturing time can lead to disease progression between leukapheresis and CAR T-cell infusion [37]. The development of allogeneic off-the-shelf CAR T cells with reduced risk of graft-versus-host disease could significantly change the workflow of CAR T-cell therapy if these treatments become available to patients without the requirements for standard CAR T-cell manufacturing [84–88]. Another potential drawback of CAR T-cell therapy is the use of preconditioning lymphodepletion regimens. Though lymphodepletion is an important part of the CAR T-cell treatment process, reduction of endogenous lymphocyte levels and subsequent CAR T-cell expansion may have implications for salvage therapy after failure of CAR T-cell therapy, as these processes modify the characteristics of patients’ T cells [87]. As a result, treatment responses to subsequent lines of therapy could be altered in these patients, and the implications of lymphodepletion regimens for treatment sequencing should be considered.

Other unique BCMA-targeted therapies are being investigated for treatment of MM. These include an anti-BCMA mAb conjugated to an antitumor maytansine derivative via a non-cleavable linker (AMG 224, under clinical study); combination therapy with an antibody-coupled T-cell receptor (ACTR087) plus an anti-BCMA antibody (SEA BCMA); a BCMA- and CD16A-directed tetravalent antibody that engages natural killer cells (AFM26); anti-BCMA recombinant immunotoxins; a heteroclitic BCMA peptide encapsulated nanoparticle-based cancer vaccine; and an antibody-based scaffold that binds CD3, BCMA, and programmed cell death ligand 1 [89–93]. Antimyeloma therapies targeting or incorporating APRIL, the primary ligand for BCMA, have also been developed. These therapies include two APRIL-based CAR T-cell constructs (ACAR, APRIL-CAR), which use truncated forms of APRIL as the tumor-targeting domain for dual targeting of the APRIL receptors BCMA and transmembrane activator and calcium-modulating cyclophilin ligand [94, 95].

In current clinical trials, patients who are treated with a previous anti-BCMA-directed therapy are often excluded from receiving any subsequent anti-BCMA treatments. Because these exclusion criteria may limit BCMA-targeted treatment options for these patients, trials assessing anti-BCMA therapies should carefully consider patient selection until we have a greater biological and clinical understanding of how anti-BCMA treatment sequencing may be conducted in the future. For example, patients at high risk of progression may not be suitable for the lag time required for CAR T-cell manufacturing and may be better suited for readily available anti-BCMA products [86]. Further assessment of anti-BCMA therapies in patients with MM with unmet needs (e.g., patients with high-risk MM, elderly and frail patients, or patients with renal failure) is also necessary, as these patients are often excluded from clinical trials [96, 97]. There are currently >50 ongoing clinical trials assessing BCMA-targeted therapies for MM, including ~15 phase 2 studies, and these trials will help gain insight into the efficacy and safety across MM populations. Furthermore, several studies are underway to assess whether combination of anti-BCMA therapies in combination with other treatments with different targets and mechanisms of action can enhance the efficacy of antimyeloma treatment regimens [7, 14, 20, 26, 27, 36–38, 94, 98–100].

Though they have predominantly been investigated in an RRMM population to date, the striking data observed with BCMA-targeted therapies suggest that these therapies could be transformative for MM treatment paradigms if used in earlier lines of treatment. Currently, even the most intensive initial lines of therapy followed by stem cell transplantation has resulted in limited extension of PFS, which necessitates the use of maintenance therapies for a prolonged duration until progression occurs [101]. If BCMA-targeted therapies are able to demonstrate deep and durable responses after short treatment durations, they may reduce the need for “treat-to-progression” paradigms for MM, which are less feasible in the real-world setting compared with clinical studies, or could even replace stem cell transplantation as first-line treatment for newly diagnosed MM, for which not all patients are eligible [31, 102].

Though mechanisms of failure of BCMA-targeted therapy are not fully known, observations and hypotheses regarding potential limitations of this approach have been reported. Targeted immunotherapies, including BCMA-targeted agents, may be affected by antigen-escape mechanisms. sBCMA levels have been widely demonstrated to decrease during treatment in response to new MM therapies, including BCMA-targeted therapies [7, 27, 36]. Although these reductions in sBCMA levels may lessen concerns with sBCMA interfering with BCMA-targeted therapies by competing with membrane-bound BCMA [38], there may also be a corresponding decline in membrane-bound BCMA that would alter the ability of BCMA-directed therapies to target MM. Indeed, a trial assessing BCMA CAR T-cell therapy observed that the majority of patients showed a decline in BCMA intensity post infusion, though membrane BCMA expression increased back toward baseline in the majority of these patients [37]. Though clinical data are limited, BCMA-negative relapse has also been reported with BCMA-targeted CAR T-cell therapy [26, 98]. Because the majority of relapses after BCMA-targeted therapies may involve BCMA-positive disease [26], retreatment with different BCMA-targeted therapies may also be feasible in the future, though sequencing with these therapies has yet to be investigated in clinical trials. Immunogenicity to anti-BCMA mAbs or scFvs could also limit the efficacy and persistence of BCMA-targeted therapy. This may be partially addressed by the use of humanized mAbs or scFvs, which are less likely to be immunogenic compared with fragments generated from other species (e.g., mice) [100]. Moreover, structural alterations have been pursued for CAR T-cell therapies, in particular to promote the expansion and persistence of these therapies in vivo, and further advancements within the MM field could benefit from similar optimization [103]. Ultimately, clinical data from larger randomized trials are needed to further understand the limitations of BCMA-directed therapies, including potential differences between BCMA-targeted bispecific antibody constructs, ADCs, and CAR T-cell therapies.

## Summary

BCMA-targeted therapies have demonstrated promising and exciting clinical results in heavily pretreated patients with RRMM. Further study is warranted to investigate whether BCMA-targeted therapies could provide long-lasting responses when used in earlier lines of therapy for MM.
